# Differences in the impact of impaired glucose status on clinical outcomes in younger and older adults: Over a decade of follow-up in the Tehran lipid and glucose study

**DOI:** 10.3389/fcvm.2022.1018403

**Published:** 2022-10-31

**Authors:** Samaneh Asgari, Soroush Masrouri, Davood Khalili, Fereidoun Azizi, Farzad Hadaegh

**Affiliations:** ^1^Prevention of Metabolic Disorders Research Center, Research Institute for Endocrine Sciences, Shahid Beheshti University of Medical Sciences, Tehran, Iran; ^2^Endocrine Research Center, Research Institute for Endocrine Sciences, Shahid Beheshti University of Medical Sciences, Tehran, Iran

**Keywords:** prediabetes, age-specific, type 2 diabetes mellitus, hypertension, cardiovascular disease, mortality events

## Abstract

**Introduction:**

Studies found that the impact of dysglycemia on microvascular, macrovascular events and mortality outcomes were different between the younger vs. older population. We aimed to investigate the age-specific association of prediabetes with clinical outcomes including type 2 diabetes (T2DM), hypertension, chronic kidney disease (CKD), cardiovascular disease (CVD), and mortality.

**Materials and methods:**

A total of 5,970 Iranians (3,829 women) aged ≥30 years, without T2DM, were included. The age-specific (<60 and ≥60 years; minimum *p*-value for interaction = 0.001) multivariable-adjusted Cox regression was done to calculate the hazard ratios (HRs) and 95% confidence intervals (CIs) of the impaired glucose status including impaired fasting glucose (IFG) vs. normal fasting glucose (NFG), impaired glucose tolerance (IGT) vs. normal glucose tolerance (NGT), and IFG&IGT vs. NFG/NGT with each outcome.

**Results:**

Among individuals aged ≥60 years, the prevalence of impaired glucose status (IFG, IGT, or both) was about 2 times higher compared to those aged <60. Age-specific association between prediabetes and incident hypertension was found for those aged <60 years; [HR (95% CI); IFG: 1.38 (1.16–1.65), IGT: 1.51 (1.26–1.81), and IFG&IGT: 1.62 (1.21–2.12)]. For CVD, in all impaired glycemic states, those aged <60 were at higher significant risk [IFG: 1.39 (1.09–1.77), IGT: 1.53 (1.19–1.97), and IFG&IGT: 1.60 (1.14–2.25)]. Stratified analyses showed similar associations for IFG and IGT with non-CV mortality 1.71 (1.04–2.80) and 2.12 (1.30–3.46), respectively, and for all-cause mortality among those aged <60 years [IFG: 1.63 (1.08–2.45) and IGT: 1.82 (1.20–2.76)]. In both age groups, all glycemic status groups were significantly associated with T2DM but not with CKD and CV mortality.

**Conclusions:**

The high prevalence of prediabetes particularly among the elderly population, limited resources, and the observed significant age differences in the impact of prediabetes states on different clinical outcomes calls for multicomponent intervention strategies by policy health makers, including lifestyle and possible pharmacological therapy, with the priority for the young Iranian population.

## Introduction

Prediabetes, typically defined as glucose concentrations higher than normal and below the current diagnostic threshold for diabetes, roughly affects 38% of US adults, and its prevalence increases with age, reaching about half of the adults aged ≥65 years (1, 2). According to nationwide epidemiological data, one in four adults aged 35–70 years in Iran live with prediabetes (3). Despite the high prevalence of prediabetes in the elderly, the population is not quite well-studied in the literature on this condition and the presentation of its related complications (4, 5).

Studies found that the impact of dysglycemia on macrovascular events and mortality outcomes were different between the younger vs. older population. The INTERHEART study conducted among 52 countries showed that the effect of dysglycemia [as assessed by glycated hemoglobin (HbA1c)] on the excess risk of myocardial infarction (MI) was more pronounced among younger individuals than older ones (6). This issue was further supported by a stratified meta-analysis conducted by Kodama et al. (7), which showed the association of both fasting plasma glucose (FPG) and 2-h post-challenge plasma glucose (2 h-PCG) with cardiovascular disease (CVD) events tended to be more prominent among younger participants. In contrast, the recent meta-analysis by Cai et al. (8) found that the unfavorable impact of prediabetes on CVD outcomes did not differ significantly between those aged ≥60 years and those younger; however, the younger group were more prone to all-cause mortality events. Additionally, some studies have reported no risk for mortality or CVD outcomes among older adults with prediabetes compared to those with normoglycemia (9–11). Regarding microvascular complications, most studies investigating the impact of prediabetes on clinical outcomes did not address the effect modification of age for chronic kidney disease (CKD) (12) and hypertension (13–15), or no interaction in this regard was found (16, 17). According to a recent umbrella review conducted by Schlesinger et al. (18), moderate certainty exists regarding the risk of prediabetes for diabetes complications, including CVD, CKD, and mortality.

Previously we assessed the sex-specific clinical outcomes of impaired glycemic states (19); in the current study, we aimed to extend our previous research by investigating the association of prediabetes, based on FPG and 2 h-PCG levels, with incident diabetes, hypertension, CKD, CVD, and mortality events over more than a decade of follow-up among older adults (aged ≥60 years) vs. younger ones.

## Materials and methods

### Study design

The Tehran Lipid and Glucose Study (TLGS) is a community-based prospective cohort study on a Tehranian urban population aged ≥3 years, which was established initially with the main objectives of determining the prevalence and incidence of non-communicable diseases (NCDs) and related risk factors. Recruitment to the study was completed in two phases, including the first (1999 to 2002; *n* = 15,005) and the second (2002 to 2005; *n* = 3,550), and follow-ups are planned for at least 20 years with a tri-annual interval design (i.e., third phase: 2005 to 2008, fourth phase: 2009 to 2011, fifth phase: 2012 to 2015, and sixth phase: 2015 to 2018). The design and methodology of the TLGS have been reported elsewhere (20). The current study included 9,747 participants, 8,071 from phase 1 and 1,676 from phase 2, aged ≥30 years.

### Study population

**Figure 1:**
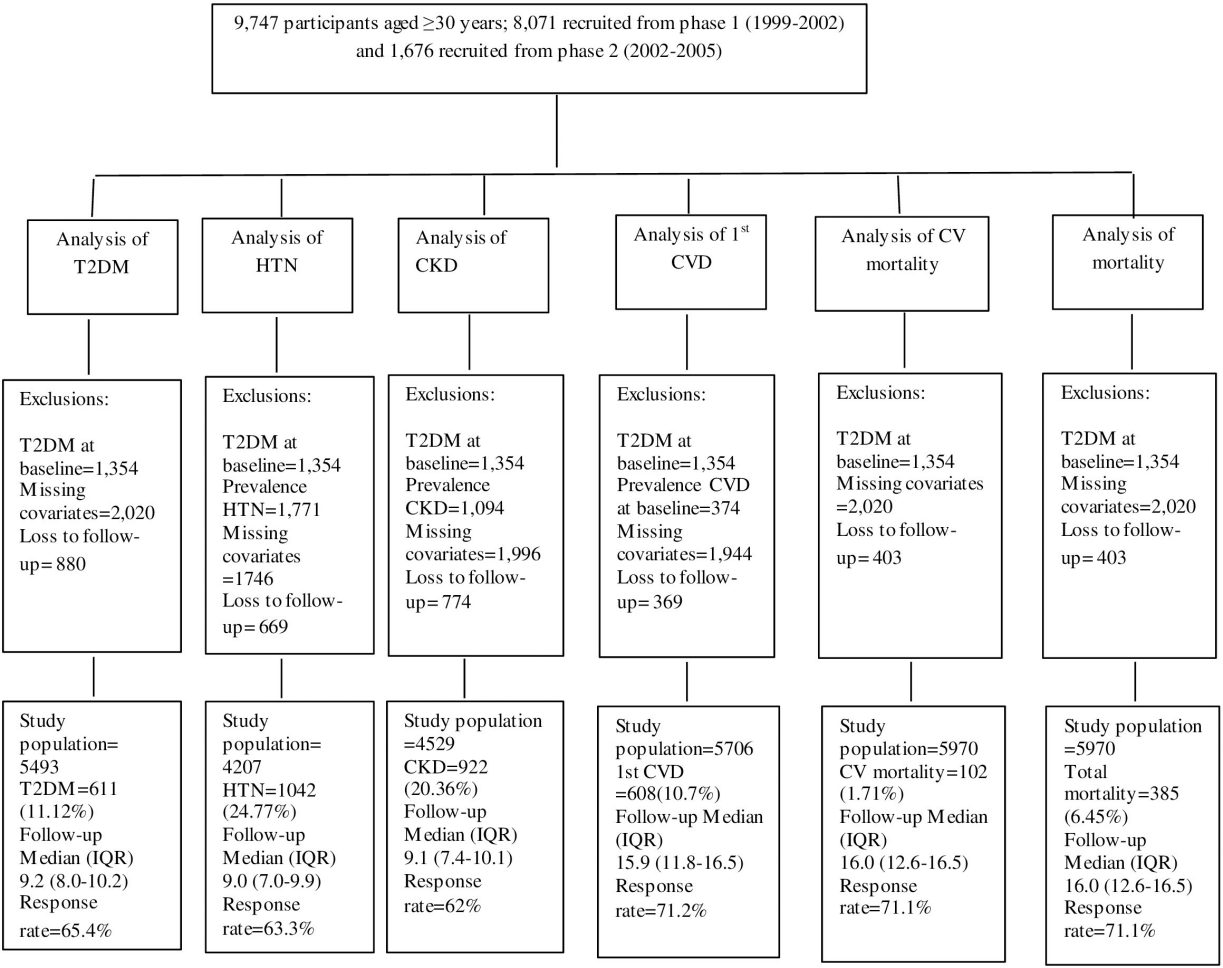
Flow diagram of the study participants. TLGS, Tehran lipids and glucose study.

Figure 1 illustrates the detailed selection process of the study population for each outcome separately. Of the total population, individuals with prevalent type 2 diabetes mellitus (T2DM) at baseline (*n* = 1,354), were excluded, leaving 8,393 individuals. Then, for the analysis of each outcome, separate exclusion criteria were applied. Therefore, for the analysis of T2DM, after excluding subjects with missing baseline values of any covariates that were used in the T2DM model (*n* = 2,020), and those with no follow-up measurements after baseline (*n* = 880), 5,493 participants remained. For the hypertension outcome, after excluding those with prevalent hypertension at baseline (*n* = 1,771), missing covariates (*n* = 1,746), or no follow-up (*n* = 669), 4,207 participants remained. For the CKD outcome, after excluding those with prevalent CKD at baseline (*n* = 1,094), missing covariates (*n* = 1,996), or no follow-up (*n* = 774), 4,529 participants remained. For the CVD outcome, after excluding those with prevalent CVD at baseline (*n* = 374), missing covariates (*n* = 1,944), or no follow-up (*n* = 369), 5,706 participants remained. For CV, non-CV, and total mortality after excluding subjects with missing covariates (*n* = 2,020), or no follow-up (*n* = 403), 5,970 participants remained.

The Institutional Review Board (IRB) of the Research Institute for Endocrine Sciences (RIES), Shahid Beheshti University of Medical Sciences, Tehran, Iran, approved this study. All participants provided written informed consent.

### Clinical and laboratory measurements

Participants were interviewed by a trained nurse, and information regarding demographics, family history of diabetes (FH-DM), history of CVD, medication history, and smoking status were collected using a standard questionnaire. Weight (kilograms) was measured to the nearest 100 grams while wearing light clothing and with shoes removed. Height (centimeters) was measured in a standing position using a tape measure, while shoulders were in normal alignment.

The participant's blood pressure was measured after a 15-min resting in a sitting position, twice on the right arm at a 5-min interval with a standardized mercury sphygmomanometer (calibrated by the Iranian Institute of Standards and Industrial Researches). The mean of the two measurements was recorded as the person's blood pressure.

A blood sample for laboratory parameters, including FPG, 2 h-PCG, creatinine (Cr), and total cholesterol (TC), was taken between 7:00 and 9:00 am from all study participants after 12 to 14 h of overnight fasting. All the blood analyses were carried out at the TLGS research laboratory on the same day of blood collection. FPG and 2 h-PCG (only among those not on glucose-lowering medications) were measured using an enzymatic colorimetric method with glucose oxidase; inter-and intra-assay coefficients of variation were both < 2.3% at baseline and follow-up phases. TC was assayed using the enzymatic colorimetric method with cholesterol esterase and cholesterol oxidase. Both inter-and intra-assay coefficients of variation were 1.9% for TC in baseline assays. Serum Cr levels were assayed by kinetic colorimetric Jaffe. Analyses were performed using Pars Azmon kits (Pars Azmon Inc., Tehran, Iran) and a Selectra 2 auto-analyzer (Vital Scientific, Spankeren, Netherlands). All samples were analyzed when internal quality control met the acceptable criteria (21). For this study, the estimated glomerular filtration rate (eGFR) was calculated using the abbreviated prediction equation, which was provided by the Chronic Kidney Disease Epidemiology Collaboration (CKD-EPI) equation (22).

### Variable definition

Body mass index (BMI; kg/m^2^) was defined as the weight (kilograms) divided by squared height (meters). Individuals who smoked cigarettes daily or occasionally were considered current smokers. Hypercholesterolemia was defined as serum TC ≥200 mg/dl or using lipid-lowering medications (23). Prevalent CVD was defined as a history of acute coronary syndrome leading to CCU admission, past history of percutaneous coronary intervention (PCI), or coronary artery bypass graft (CABG), angiographic proven coronary artery disease (i.e., >50% luminal narrowing of one or more coronary artery), or history of stroke events. Based on the American Diabetes Association, glycemic categories among those without T2DM were defined as impaired fasting glucose (IFG): FPG range of 100–125 mg/dl; impaired glucose tolerance (IGT): 2 h-PCG range of 140–200 mg/dl; and both IFG and IGT (IFG&IGT) (24). In the current study, individuals within the age range ≥60 years are classified as elderly; those aged 30–60 years are classified as young/middle-aged adults.

### Outcome definitions

T2DM was defined as FPG ≥126 mg/dl, or 2 h-PCG ≥200 mg/dl, or taking anti-diabetes medication.

Hypertension was defined as systolic blood pressure (SBP) ≥140 mmHg, diastolic blood pressure (DBP) ≥90 mmHg, or using antihypertensive medications.

CKD was considered an eGFR below than 60 ml/min/1.73 m^2^.

For the collection of CVD and mortality, each participant was followed up annually for any medical event leading to hospitalization; a trained interviewer asked participants regarding any related medical condition, and a trained physician collected complementary data for that event during a home visit and by acquiring data from medical files from hospitals and any medical encounters. Collected data were evaluated by an outcome committee (Cohort Outcome Panel) consisting of a principal investigator, an internist, an endocrinologist, a cardiologist, an epidemiologist, and the physician who collected the outcome data; other experts were invited as required for the evaluation of non-communicable disorders. The final diagnosis was adjudicated by consensus of the majority of committee members (i.e., by ≥3 members of the committee) (25). In the current study, incident CVD event was defined as definite myocardial infarction (MI), probable MI, unstable angina, angiographic-proven coronary heart disease (CHD), and stroke (defined by a new neurological deficit that lasted more than 24 h).

In the case of mortality, data were collected by an authorized local physician from the hospital or the death certificate.

### Statistical analysis

Baseline characteristics of the study population were described as mean ± standard deviation (SD) values for continuous variables and frequencies (%) for categorical variables across glycemic categories for each age group (< 60 and ≥60 years) separately. The baseline characteristics of the participants across glycemic categories were compared using the student's *t*-test for normally-distributed continuous variables, the Chi-squared test for categorical variables, and the Mann-Whitney U statistic for skewed and ordered variables.

To evaluate the association of glycemic categories (IFG, IGT, and IFG&IGT) with the incident of each outcome, Cox proportional hazard models were applied; model 1: was adjusted with age and sex; model 2 was further adjusted with BMI, eGFR, current smoking, hypercholesterolemia, FH-DM (only for incident T2DM), prevalent CVD (except for incident 1st CVD), and hypertension (except for incident hypertension). The adjusted hazard ratios (HRs) and 95% confidence intervals (CI) were reported for each of the IFG, IGT, and IFG&IGT categories, considering the normal fasting glucose (NFG), normal glucose tolerance (NGT), and NFG/NGT as reference categories, respectively. The event date for incident T2DM, hypertension, and CKD cases was described as the mid-time between the date of the follow-up visit at which each outcome was detected for the first time and the most recent follow-up visit preceding the diagnosis; the follow-up time was drawn from the difference between the calculated mid-time date and the date at which the subjects entered the study. For the censored and lost follow-up individuals, the survival time was the interval between the first and the last observation dates. The proportionality in the Cox models was evaluated with the Schoenfeld residual test; generally, all proportionality assumptions were appropriate.

We evaluated the effect modification of gender and age for different glycemic categories in a multivariable model. Since significant interactions were observed between age groups and different glycemic categories (min *p*-value =0.001), the analyses were performed in each age group separately. For the interaction *p*-value, we did not consider the multiple Bonferroni correction tests for two reasons: first, to reduce the chance of obligating type II errors, and second, the comparison was made for each glycemic category independently (26, 27). No interaction was observed between gender and glycemic categories; therefore, gender was adjusted in the models. All analyses were conducted using STATA version 14 SE (StataCorp, TX, USA), and a two-tailed *p*-value <0.05 was considered significant.

## Results

### Baseline characteristics

**Figure 2:**
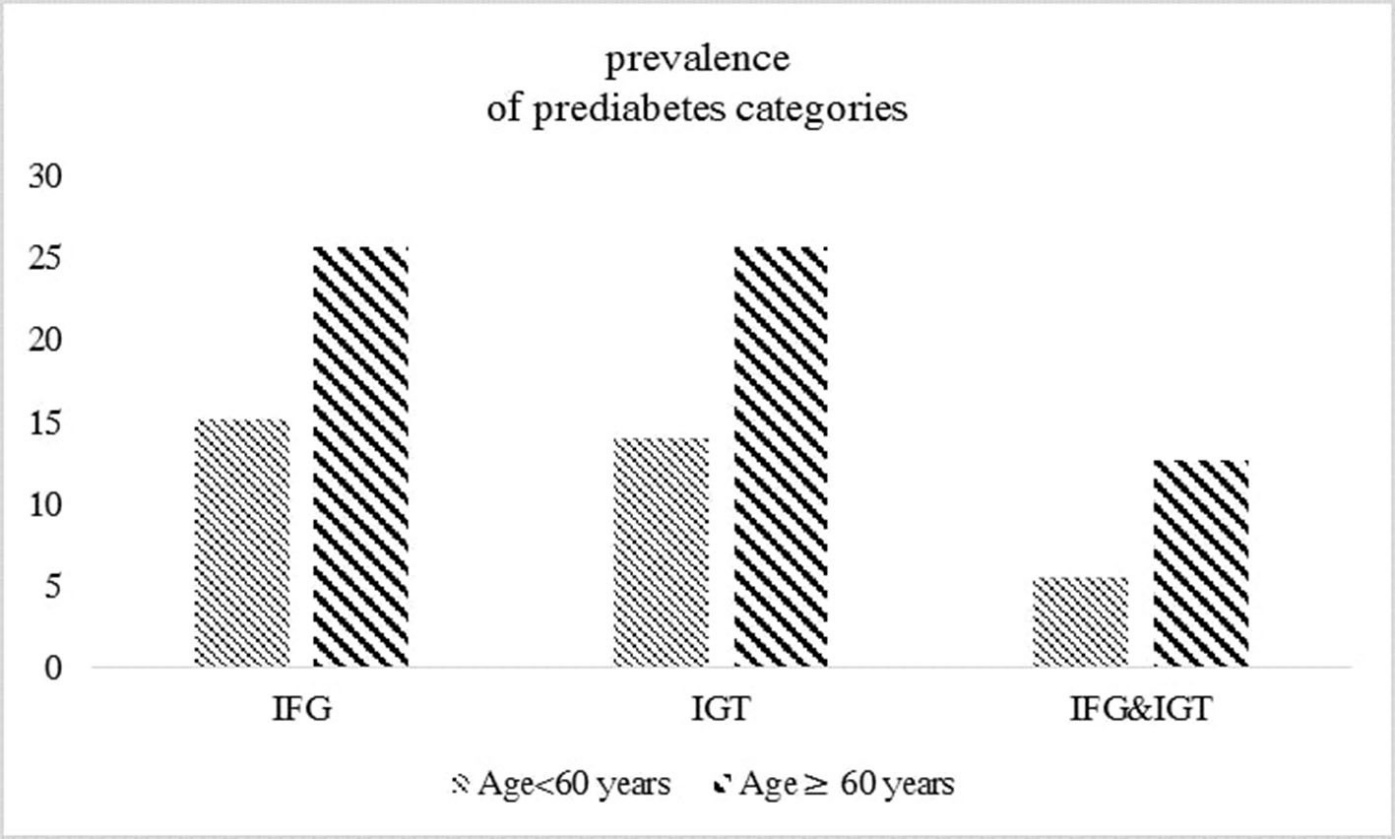
The prevalence of prediabetes categories in the overall sample (*n* = 5970) stratified by age groups.

The study population consists of 5,970 participants, including 1,040 participants aged ≥60 years (mean ± SD: 66.27 ± 5.19) and 4,930 participants aged <60 years (mean ± SD: 41.96 ± 8.24). Baseline characteristics according to glycemic categories for each age group are shown in Table 1. Participants with impaired glycemic status (IFG, IGT, or both) had higher BMI, FPG, and 2 h-PCG levels across all glycemic categories in both age groups. Among participants aged <60 years, those with impaired glucose status generally had lower eGFR, higher SBP, DBP, TC levels, and higher CVD prevalence than the corresponding reference categories. Among those aged ≥60 years, compared to participants with NFG or NGT, BMI, SBP, and TC levels were higher among those with each IFG and IGT. Also, compared to those with NGT, individuals with IGT had higher BMI, SBP, DBP, and TC levels. The prevalence of prediabetes categories in the overall sample stratified by age categories is represented in Figure 2. Among individuals aged ≥60 years, the prevalence of impaired glucose status (IFG, IGT, or both) was about two times higher compared to those aged <60 years.

**Table 1:** Baseline characteristics of the study participants (*N* = 5,970) according to the prediabetes categories: Tehran lipid and glucose study 1999–2018.

**Prediabetes categories**		**FPG, mg/dl**					**2 h–PCG, mg/dl**				**FPG and 2 h–PCG, mg/dl**	
	**Total**	**<100**	**100–125**	***p*** –value^*^		**<140**	**140–200**	***p*** –value^*^		**<100 or <140**	**100–125&140–199**	***p*** –value^*^
**Age <60 years**												
Population, n	4,930	4,182	748			4,237	693			4,657	273	
Age, years	41.96 ± 8.24	41.37 ± 8.14	45.24 ± 8.03	<0.0001		41.41 ± 8.16	45.28 ± 7.91	<0.0001		41.68 ± 8.19	46.62 ± 7.61	<0.0001
Gender, female	3,289 (66.71)	2,815 (67.31)	474 (63.37)	0.035		2,774 (65.47)	515 (74.31)	<0.0001		3,093 (66.42)	196 (71.79)	0.067
BMI, kg/m^2^	27.62 ± 4.58	27.32 ± 4.43	29.26 ± 5.00	<0.0001		27.31 ± 4.50	29.49 ± 4.62	<0.0001		27.47 ± 4.51	30.15 ± 4.97	<0.0001
eGFR, ml/min/1.73 m^2^	75.26 ± 12.11	75.70 ± 12.08	72.80 ± 11.95	<0.0001		75.75 ± 12.09	72.24 ± 11.81	<0.0001		75.47 ± 12.08	71.57 ± 12.05	<0.0001
SBP, mmHg	116.25 ± 16.07	114.97 ± 15.35	123.41 ± 18.00	<0.0001		115.00 ± 15.35	123.96 ± 18.08	<0.0001		115.65 ± 15.69	126.53 ± 18.78	<0.0001
DBP, mmHg	77.61 ± 10.34	76.98 ± 10.22	81.18 ± 10.29	<0.0001		76.91 ± 10.08	81.92 ± 10.88	<0.0001		77.33 ± 10.26	82.48 ± 10.57	<0.0001
TC, mg/dl	208.98 ± 43.77	207.02 ± 43.41	219.96 ± 44.17	<0.0001		206.35 ± 43.00	225.06 ± 45.13	<0.0001		207.79 ± 43.34	229.34 ± 46.04	<0.0001
FPG, mg/dl	90.13 ± 9.55	87.27 ± 6.89	106.13 ± 5.78	<0.0001		89.03 ± 8.76	96.87 ± 11.23	<0.0001		89.08 ± 8.60	108.08 ± 6.57	<0.0001
2 h–PCG, mg/dl	108.55 ± 28.86	104.88 ± 26.46	128.80 ± 32.92	<0.0001		100.14 ± 20.82	159.33 ± 15.46	<0.0001		105.26 ± 25.94	164.15 ± 16.12	<0.0001
Prevalent CVD, yes	136 (2.76)	106 (2.53)	30 (4.01)	0.023		100 (2.36)	36 (5.19)	<0.0001		119 (2.56)	17 (6.23)	<0.0001
Hypertension, yes	862 (17.48)	708 (16.93)	154 (20.59)	0.015		712 (16.80)	150 (21.65)	0.002		801 (17.20)	61 (22.34)	0.030
Hypercholesterolemia, yes	2,737 (55.52)	2,241 (53.59)	496 (66.31)	<0.0001		2,255 (53.22)	482 (69.55)	<0.0001		2,538 (54.50)	199 (72.89)	<0.0001
Current smoking, yes	687 (13.94)	592 (14.16)	95 (12.70)	0.290		624 (14.73)	63 (9.09)	<0.0001		659 (14.15)	28 (10.26)	0.071
**Age ≥60 years**												
Population, *n*	1,040	773	267			772	268			908	132	
Age, years	66.27 ± 5.19	66.39 ± 5.34	65.94 ± 4.70	0.218		66.24 ± 5.38	66.36 ± 4.61	0.748		66.31 ± 5.30	66.00 ± 4.34	0.516
Gender, female	540 (51.92)	405 (52.39)	135 (50.56)	0.606		399 (51.68)	141 (52.61)	0.793		475 (52.31)	65 (49.24)	0.509
BMI, kg/m^2^	27.12 ± 4.45	26.77 ± 4.46	28.14 ± 4.26	<0.0001		26.82 ± 4.37	27.98 ± 4.57	<0.001		26.93 ± 4.41	28.38 ± 4.47	<0.001
eGFR, ml/min/1.73 m^2^	60.05 ± 10.72	60.33 ± 10.76	59.24 ± 10.61	0.154		60.24 ± 10.82	59.50 ± 10.45	0.333		60.17 ± 10.76	59.21 ± 10.45	0.337
SBP, mmHg	135.69 ± 21.68	135.01 ± 22.12	137.67 ± 20.26	0.084		134.07 ± 21.74	140.38 ± 20.86	<0.0001		134.88 ± 21.59	141.27 ± 21.60	0.002
DBP, mmHg	80.34 ± 12.05	80.09 ± 12.14	81.07 ± 11.78	0.253		79.77 ± 12.06	82.00 ± 11.89	0.009		80.09 ± 12.00	82.11 ± 12.41	0.071
TC, mg/dl	226.17 ± 44.73	224.72 ± 43.61	230.39 ± 47.68	0.074		223.87 ± 42.83	232.79 ± 49.30	0.005		224.80 ± 43.50	235.65 ± 51.64	0.009
FPG, mg/dl	93.44 ± 10.04	88.84 ± 6.40	106.73 ± 6.00	<0.0001		91.41 ± 9.04	99.29 ± 10.48	<0.0001		91.32 ± 8.63	107.97 ± 6.28	<0.0001
2h–PCG, mg/dl	120.89 ± 30.72	115.14 ± 28.08	137.06 ± 32.09	<0.0001		106.68 ± 20.74	160.60 ± 15.44	<0.0001		114.51 ± 27.00	163.68 ± 16.75	<0.0001
Prevalent CVD, yes	131 (12.60)	98 (12.68)	33 (12.36)	0.892		96 (12.44)	35 (13.06)	0.791		111 (12.22)	20 (15.15)	0.344
Hypertension, yes	223 (21.44)	167 (21.60)	56 (20.97)	0.830		180 (23.32)	43 (16.04)	0.012		197 (21.70)	26 (19.70)	0.601
Hypercholesterolemia, yes	749 (72.02)	550 (71.15)	199 (74.53)	0.289		545 (70.60)	204 (76.12)	0.083		647 (71.26)	102 (77.27)	0.150
Current smoking, yes	104 (10.00)	85 (11.00)	19 (7.12)	0.068		79 (10.23)	25 (9.33)	0.671		92 (10.13)	12 (9.09)	0.709

SD, standard deviation; BMI, body mass index; SBP, systolic blood pressure; DBP, diastolic blood pressure; FPG, fasting plasma glucose; TC, total cholesterol; 2h–PCG, 2–h post–challenge plasma glucose.

Values are shown as mean ± SD and number (%), for continuous and categorical variables, respectively.

* The comparison *p*–value between groups was calculated using a *t–*test for normal continuous variables, and a chi–square test (fisher's exact test if required) for categorical variables.

**Figure 3:**
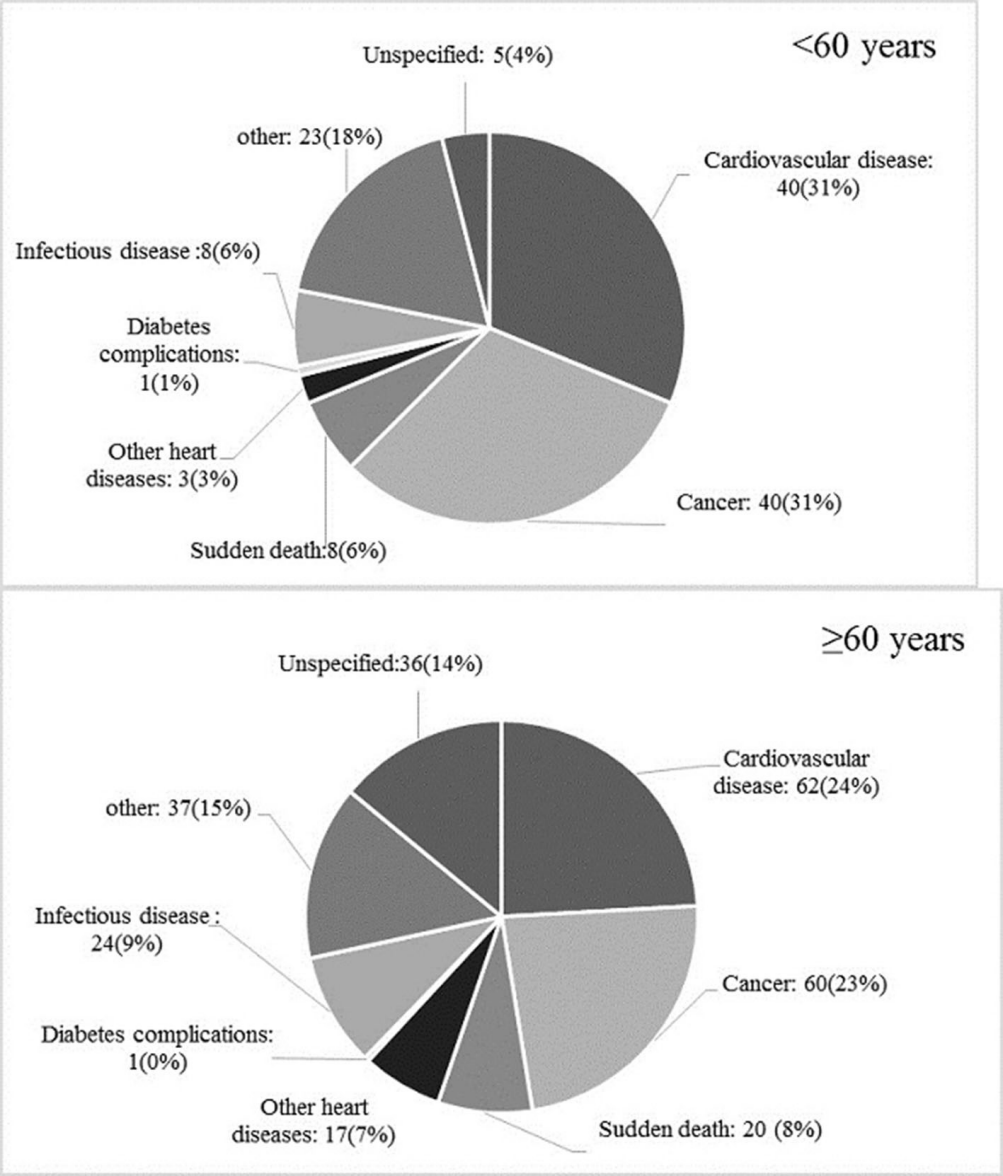
Age-specific distribution of different causes of total mortality among the study population.

During a study period, 385 deaths (257 among those aged ≥60 years) have been recorded. Age-specific distribution of different reasons for death is shown in Figure 3. Underlying causes of mortality in adults aged <60 years were CVD (*n* = 40), cancer (*n* = 40), sudden death (*n* = 8), infectious diseases (*n* = 8), other heart diseases (e.g. heart valve replacement) (*n* = 3), diabetes complications (*n* = 1), others (e.g. accident, poisoning) (*n* = 23), and without classified cause (*n* = 5). Among individuals aged ≥60 years, specific causes of mortality were CVD (*n* = 62), cancer (*n* = 60), sudden death (*n* = 20), infectious diseases (*n* = 24), other heart diseases (*n* = 17), diabetes complications (*n* = 1), and others (*n* = 37). Moreover, 36 cases of death had not a classified cause. The multivariable-adjusted HRs (95% CI) of the impaired glucose status across glycemic categories (IFG vs. NFG, IGT vs. NGT, IFG&IGT vs. NFG/NGT), in association with different outcomes for each age category, are shown in Tables 2, 3.

**Table 2:** Adjusted hazard ratios (95 % CI) for incident T2DM, HTN, and CKD by age groups: Tehran lipid and glucose study.

	**Age <60 years**				**Age ≥60 years**			
	**E/*N***	**Model 1**	**Model 2**		**E/N**	**Model 1**	**Model 2**	**Interaction**
		**HR (95 % CI)**	**HR (95 % CI)**			**HR (95 % CI)**	**HR (95 % CI)**	***p*** –value^*^
**Incident T2DM**								
FPG <100 mg/dl	380/4,400	Reference	Reference		110/877	Reference	Reference	0.037
FPG (100–125 mg/dl)	91/151	**7.51 (6.27–9.00)**	**6.02 (5.00–7.26)**		30/65	**4.35 (3.11–6.08)**	**4.02 (2.87–5.62)**	
2 h–PCG <140 mg/dl	247/3,933	Reference	Reference		59/697	Reference	Reference	0.23
2 h–PCG (140–199 mg/dl)	224/618	**7.07 (5.90–8.47)**	**5.70 (4.73–6.87)**		81/245	**4.89 (3.49–6.84)**	**4.51 (3.22–6.32)**	
FPG <100 mg/dl or 2 h–PCG <140 mg/dl	339/4,315	Reference	Reference		83/819	Reference	Reference	0.15
FPG (100–125 mg/dl) and 2 h–PCG (140–199 mg/dl)	132/236	**10.52 (8.60–12.89)**	**7.95 (6.44–9.81)**		57/123	**6.58 (4.70–9.23)**	**5.96 (4.24–8.36)**	
**Incident HTN**								
FPG <100 mg/dl	800/3,656	Reference	Reference		201/429	Reference	Reference	0.047
FPG (100–125 mg/dl)	29/100	**1.64 (1.37–1.95)**	**1.38 (1.16–1.65)**		12/22	1.09 (0.80–1.50)	0.96 (0.70–1.32)	
2 h–PCG <140 mg/dl	685/3,337	Reference	Reference		173/357	Reference	Reference	0.001
2 h–PCG (140–199 mg/dl)	144/419	**1.84 (1.54–2.20)**	**1.51 (1.26–1.81)**		40/94	0.80 (0.56–1.12)	0.76 (0.54–1.07)	
FPG < 100 mg/dl or 2 h–PCG <140 mg/dl	770/3,601	Reference	Reference		189/406	Reference	Reference	0.06
FPG (100–125 mg/dl) and 2 h–PCG (140–199 mg/dl)	59/155	**2.02 (1.55–2.64)**	**1.62 (1.21–2.12)**		24/45	1.06 (0.69–1.63)	1.00 (0.65–1.53)	
**Incident CKD**								
FPG <100 mg/dl	675/3,954	Reference	Reference		200/412	Reference	Reference	0.50
FPG (100–125 mg/dl)	30/133	**1.39 (1.15–1.69)**	1.13 (0.93–1.38)		17/30	1.19 (0.88–1.60)	1.00 (0.74–1.35)	
2 h–PCG <140 mg/dl	588/3,543	Reference	Reference		161/328	Reference	Reference	0.33
2 h–PCG (140–199 mg/dl)	117/544	1.25 (0.73–1.33)	0.97 (0.79–1.19)		56/114	0.98 (0.73–1.33)	0.81 (0.60–1.10)	
FPG <100 mg/dl or 2 h–PCG <140 mg/dl	659/3,880	Reference	Reference		187/387	Reference	Reference	0.65
FPG (100–125 mg/dl) and 2 h–PCG (140–199 mg/dl)	46/207	1.34 (0.99–1.80)	1.01 (0.74–1.36)		30/55	1.06 (0.72–1.56)	0.90 (0.61–1.33)	

FPG, fasting plasma glucose; 2 h–PCG, 2–h post–challenge plasma glucose; HR, hazard ratio; T2DM, type 2 diabetes mellitus; HTN, hypertension; CKD, chronic kidney disease.

Model 1, adjusted for age, and gender; model 2, adjusted for age, gender, body mass index, hypercholesterolemia, hypertension, current smoking, eGFR, prevalent CVD.

Bold values are statistically significant.

**Table 3:** Adjusted hazard ratios (95 % CI) for incident CVD, CV mortality, and total mortality by age groups: Tehran lipid and glucose study.

	**Age <60 years**				**Age ≥60 years**			
	**E/N**	**Model 1**	**Model 2**		**E/N**	**Model 1**	**Model 2**	**Interaction**
		**HR (95 % CI)**	**HR (95 % CI)**			**HR (95 % CI)**	**HR (95 % CI)**	p–value^*^
**Incident 1st CVD**								
FPG <100 mg/dl	343/4,627	Reference	Reference		222/850	Reference	Reference	0.31
FPG (100–125 mg/dl)	26/167	**1.77 (1.39–2.25)**	**1.39 (1.09–1.77)**		17/62	1.29 (0.98–1.70)	1.15 (0.87–1.52)	
2 h–PCG <140 mg/dl	287/4,137	Reference	Reference		171/679	Reference	Reference	0.037
2 h–PCG (140–199 mg/dl)	82/657	**2.01 (1.57–2.58)**	**1.53 (1.19–1.97)**		68/233	1.21 (0.92–1.61)	1.03 (0.77–1.36)	
FPG <100 mg/dl or 2 h–PCG < 140 mg/dl	331/4,538	Reference	Reference		204/800	Reference	Reference	0.062
FPG (100–125 mg/dl) and 2 h–PCG (140–199 mg/dl)	38/256	**2.24 (1.60–3.13)**	**1.60 (1.14–2.25)**		35/112	1.21 (0.84–1.73)	1.00 (0.70–1.43)	
**CV mortality**								
FPG <100 mg/dl	30/4,182	Reference	Reference		50/773	Reference	Reference	0.09
FPG (100–125 mg/dl)	10/748	1.81 (0.89–3.71)	1.46 (0.71–3.01)		12/267	0.67 (0.36–1.27)	0.64 (0.34–1.20)	
2 h–PCG <140 mg/dl	32/4,237	Reference	Reference		46/772	Reference	Reference	0.49
2 h–PCG (140–199 mg/dl)	8/693	1.65 (0.76–3.58)	1.26 (0.57–2.76)		16/268	1.04 (0.59–1.83)	0.90 (0.50–1.59)	
FPG <100 mg/dl or 2 h–PCG <140 mg/dl	37/4,657	Reference	Reference		59/908	Reference	Reference	0.14
FPG (100–125 mg/dl) and 2 h–PCG (140–199 mg/dl)	3/273	1.46 (0.45–4.74)	1.00 (0.30–3.27)		3/132	0.34 (0.11–1.01)	**0.29 (0.09–0.93)**	
**Non–CV mortality**								
FPG <100 mg/dl	64/4,182	Reference	Reference		146/773	Reference	Reference	0.06
FPG (100–125 mg/dl)	21/748	**1.73 (1.06–2.83)**	**1.71 (1.04–2.80)**		49/267	0.96 (0.69–1.32)	0.98 (0.70–1.36)	
2h–PCG <140 mg/dl	66/4,237	Reference	Reference		140/772	Reference	Reference	0.045
2 h–PCG (140–199 mg/dl)	22/693	**2.10 (1.29–3.41)**	**2.12 (1.30–3.46)**		55/268	1.18 (0.86–1.61)	1.17 (0.86–1.61)	
FPG <100 mg/dl or 2h–PCG <140 mg/dl	80/4,657	Reference	Reference		168/908	Reference	Reference	0.27
FPG (100–125 mg/dl) and 2h–PCG (140–199 mg/dl)	8/273	1.76 (0.85–3.65)	1.72 (0.83–3.59)		27/132	1.08 (0.72–1.63)	1.08 (0.72–1.63)	
**Total mortality**								
FPG <100 mg/dl	97/4,182	Reference	Reference		196/773	Reference	Reference	0.016
FPG (100–125 mg/dl)	31/748	**1.76 (1.17–2.63)**	**1.63 (1.08–2.45)**		61/267	0.88 (0.66–1.78)	0.88 (0.66–1.18)	
2 h–PCG <140 mg/dl	98/4,237	Reference	Reference		186/772	Reference	Reference	0.044
2 h–PCG (140–199 mg/dl)	30/693	**1.95 (1.29–2.94)**	**1.82 (1.20–2.76)**		71/268	1.14 (0.87–1.50)	1.09 (0.83–1.45)	
FPG <100 mg/dl or 2 h–PCG <140 mg/dl	117/4,657	Reference	Reference		227/908	Reference	Reference	0.14
FPG (100–125 mg/dl) and 2 h–PCG (140–199 mg/dl)	11/273	1.67 (0.90–3.09)	1.47 (0.79–2.75)		30/132	0.89 (0.61–1.30)	0.85 (0.58–1.25)	

FPG, fasting plasma glucose; 2 h–PCG, 2–h post–challenge plasma glucose; HR, hazard ratio; CVD, cardiovascular disease.

Model 1, adjusted for age, and gender; model 2, adjusted for age, gender, body mass index, hypercholesterolemia, hypertension, current smoking, eGFR, prevalent CVD.

Bold values are statistically significant.

### T2DM

Regarding T2DM, all glycemic categories in model 1 were associated with at least 4.35 times increased risk among both age groups. Among the population aged <60 years, the multivariable-adjusted HRs (95% CI) of incident T2DM were 6.02 (5.00–7.26) for IFG, 5.70 (4.73–6.87) for IGT, and 7.95 (6.44–9.81) for IFG&IGT groups, compared to the related reference categories. The corresponding multivariable HRs (95% CI) for participants aged ≥60 years were 4.02 (2.87–5.62), 4.51 (3.22–6.32), and 5.96 (4.24–8.36). Among different definitions of prediabetes, only IFG was found to have a statistically significant higher impact on incident T2DM among the young/middle-aged adults vs. the elderly (*P* for interactio*n* = 0.037).

### Hypertension

As shown in Table 2, in both models, none of the groups of impaired glucose status remained a predictor of incident hypertension among people aged ≥60 years. Among those aged <60 years, model 1 showed a positive association between IFG, IGT, and IFG&IGT with the risk of hypertension. In model 2, after further adjustment, the association remained significant [1.38 (95% CI; 1.16–1.65) for IFG, 1.51 (1.26–1.81) for IGT, and 1.62 (1.21–2.12) for IFG&IGT]. In the multivariable-adjusted models, a significant interaction of age with each IFG and IGT on the risk of hypertension was observed (*P* for interactio*n* = 0.047 and 0.001, respectively).

As a sensitivity analysis we considered 2017 ACC/AHA threshold (12) (≥130/80 mmHg) for the diagnosis of hypertension. Among those aged <60 years, the results showed a higher risk of IGT for the incident hypertension in both models 1 and 2 [1.22 (95% CI; 1.02–1.47) and 1.23 (1.02–1.48), respectively]. However, we did not find any association between each glycemic category and incident hypertension based on ACC/AHA definition among elder population (Supplementary Table 1).

### CKD

IFG among people aged <60 years for incident CKD was a significant predictor only in model 1 [HR (95% CI): 1.39 (1.15–1.69)]; however, after further adjustments for potential confounders, the risk attenuated to 13% and reached null. We also did not find any association between each glycemic category and incident CKD among the population aged ≥60 years.

### CVD

For incident 1st CVD, in the age and sex-adjusted model, impaired glucose status across all three glycemic categories was significantly associated with an elevated risk among those aged <60 years [HR (95% CI) of 1.77 (1.39–2.25) for IFG, 2.01 (1.57–2.58) for IGT, and 2.24 (1.60–3.13) for IFG&IGT] (Table 3); Overall, after additional adjustments in model 2, the HRs decreased but were still significant. Moreover, impaired glucose status in all categories for those aged ≥60 years did not show any significant associations for the risk of incident 1st CVD even in model 1. Among different definitions of prediabetes, only IGT was found to have a statistically significant higher impact for incident 1st CVD among young/middle-aged adults vs. the elderly population (*P* for interactio*n* = 0.037).

### Total mortality

Considering total mortality, after adjustment for age and sex, HRs were 1.76 (95% CI; 1.17–2.63) for IFG and 1.95 (1.29–2.94) for IGT among participants aged <60 years (Table 3). IFG and IGT groups had an increased risk of total mortality by 63 and 82% in the multivariable model. Among the population aged ≥60 years, glycemic categories did not show any significant association with total mortality (*P* for interactio*n* = 0.016, and 0.044, for IFG and IGT, respectively). Yet, no significant associations between IFG&IGT with total mortality were found in either age group.

### CV mortality

The analysis did not show a significant association between glycemic categories and CV mortality in either model 1 or model 2 among the young/middle-aged adults and the elderly population.

### Non-CV mortality

As shown in Table 3, for those aged <60 years, approximately 70 and 110% increased risk of non-CV mortality for IFG and IGT was observed in model 1, respectively. After further adjustment in model 2, the HRs of non-CV mortality risk were 1.71 (95% CI; 1.04–2.80) for IFG and 2.12 (1.30–3.46) for IGT. No similar associations were found for the participants aged ≥60 years (*P* for interactio*n* = 0.06 and 0.045, respectively).

## Discussion

In this long-term population-based study among Tehranian adults, after accounting for traditional cardiovascular risk factors, we observed that prediabetes status, regardless of its definition, was significantly associated with a higher risk of CVD and mortality events among younger versus older population. Although prediabetes increased the risk of incident T2DM in both young/middle-aged and elderly adults, with a stronger impact on the former, the risk of hypertension was only present in individuals aged <60 years.

It is well-established that prediabetes is associated with an increased risk of CVD, CV, and total mortality (18); growing evidence from longitudinal cohort studies shows that the role of prediabetes might be less prominent in the elderly (10, 28, 29) or might take a different course (9, 30). In our study, prediabetes defined by IFG, IGT, or IFG&IGT was associated with a 39–60% increased risk of incident CVD in younger participants (<60 years); however, no association was observed among the elderly. Cai et al. (8), in a systematic review and meta-analysis involving 10 069 955 individuals, found that prediabetes (as measured with FPG, 2h-PCG, and HbA1_c_) was associated with a 15 and 10% increased risk of composite CVD among individuals aged <60 years and those aged ≥60 years, respectively. Furthermore, our age-stratified analysis showed no significant association between prediabetes (IFG, IGT, or IFG&IGT) and CV mortality among either age group. Despite our results, the meta-analysis of 26 prospective cohort studies (31) showed that among adults aged <55 years, IFG-ADA and IGT significantly increased the risk of CV mortality by 51 and 18%, respectively; among the population aged ≥55 years, only IGT was associated with higher risk.

We also found that IFG and IGT (but not IFG&IGT) displayed a 63 and 82% higher risk of all-cause mortality in young/middle-aged adults, respectively. However, among the older population, no significant association was found in this regard. In line with our study, Huang et al. (32) reported that IFG-ADA was significantly associated with a 28% increased risk of all-cause mortality among subjects aged <55 years but not in those aged ≥55 years (*P* for heterogeneity =0.009); they also showed that IGT significantly increased the risk of all-cause mortality by 36 and 19% among those aged <55 and ≥55 years, respectively (*P* = 0.07 for heterogeneity). In another published meta-analysis by Huang et al. (31), among adults aged <55 years, IFG-ADA (heterogeneity =85.8%), IGT (heterogeneity =54.7%), and the combination of IFG 110 mg/dl and/or IGT (heterogeneity=0%) were observed to be associated with 31, 36, and 24% increased risk of all-cause mortality, respectively; for those aged ≥55 years, IGT and the combination of IFG 110 mg/dl and/or IGT significantly increased the risk of all-cause mortality by 20%. Furthermore, the results of our study showed that the risk observed for all-cause mortality was mostly derived from non-CV mortality. IFG and IGT increased the risk of non-CV mortality by 71 and 112% among younger adults, respectively; no significant association between IFG&IGT combination in this regard was found. Together with existing evidence (28), prediabetes was not associated with non-CV mortality among those aged ≥60 years. Notably the effect sizes of glycemic categories of CVD and CV mortality are almost the same, but as a result of the relatively small sample size of the CVD mortality, the power was not enough to detect a true association between prediabetes categories and CV mortality. Different findings in elder adults might be related to the effect of insulin on CVD and CV mortality. Fasting insulin levels as a marker of Insulin resistance are shown to be associated with CVD (33, 34) among those without T2DM. A 2012 meta-analysis of 16 studies, which included 46,236 participants, demonstrated that pooled relative risk of CVD per 1-SD increase was 1.13 (95% CI: 1.05, 1.22; I^2^:58.3%) for insulin and 1.25 (95% CI: 1.16, 1.35; I^2^:52.4%) for Homeostatic Model Assessment for Insulin Resistance (HOMA-IR) (35). In another meta-analysis, with 7 articles involving 26,976 non-diabetic adults, HOMA-IR but not fasting insulin appears to be independently associated with a higher risk of CV mortality (I^2^ = 75.4%) (36). However, few studies reported the age-specific association of insulin with CVD. Data from the Prospective Study of Pravastatin in the Elderly at Risk (PROSPER) cohort showed that insulin and HOMA-IR are not associated with an increased risk of incident CVD in elderly people without diabetes (37). In another study conducted by Lu et.al (38), the association between HOAMA-IR and a person's risk of CVD using the Framingham risk score (FRS) was evaluated by Taiwanese people aged ≥50 years. They found that HOMA-IR >1.15 were significantly associated with a high level of FRS (≥ 20%) but it is not recommended to use solely for evaluating the CVD risk because of the low level of the areas under the curve (0.627). Regarding young age, the findings of the study using the Archimedes model in non-diabetic adults aged 20–30 years showed that preventing insulin resistance could avert about 42% of MI during a simulated follow-up duration of 60 years (39).

Regarding T2DM, all categories of impaired glucose status in both age groups conferred a minimum of a four-fold increase in the risk of T2DM. In line with our findings, in a previous Cochrane Database systemic review, those with prediabetes (as defined by IFG, IGT, and IFG and IGT) had a 3.50- to 7-fold higher risk of incident T2DM; as predicted, IFG and IGT was a stronger predictor compared to IFG, or IGT (16). We also found that the increased risk of T2DM was significantly more pronounced in those aged <60 years with prediabetes only when it was defined as IFG. Subsequent studies have reported mixed results in this regard; for example, Kim et al. (40), after subgroup analysis in a large cohort of 2 513 127 Korean people without diabetes, found that the effect of cumulative IFG exposure on the risk of T2DM was more prominent in those aged <65 years compared to older people. Another study from The Brazilian Longitudinal Study of Adult Health (ELSA-Brasil) among 15 105 individuals found that in participants with prediabetes, those aged 65–74 years almost always had a lower rate of conversion to T2DM than those in the 55–64 age group (41). However, an individual participant data meta-analysis of 76 513 participants showed no significant difference in the predictive ability of prediabetes for incident T2DM between those in the age categories of ≥60, 50–59, and <50 years (42). Lifestyle improvements are particularly efficient in decreasing T2DM risk; accordingly, evidence from clinical trials indicates that lifestyle interventions helped halt progression to T2DM (43). According to Diabetes Prevention Program (DPP), as highlighted in the American Diabetes Association (ADA) 2022 (44, 45), Metformin was as effective as lifestyle changes in individuals aged 25–59 years, with BMI≥35 kg/m^2^, and those with FPG >110 mg/dl.

To our knowledge, this study is the first to find effect modification of age for the risk of incident hypertension associated with IFG or IGT, showing an increased risk in young/middle-aged participants but not the elderly. While most (13, 14, 46–48) but not all previous studies (15, 49, 50) found that intermediate hyperglycemia burden confers a higher risk of incident hypertension, no study found a significant difference among age groups regarding this risk (50–52). Sasaki et al. (53) found that among 2,136 and 3,426 Japanese people aged < 65 and ≥65 years, respectively, only middle-aged participants with prediabetes (as defined by IFG or IGT) had an elevated risk for hypertension; however, among middle-aged participants, in the multivariable model, IGT showed a signal of increased risk of hypertension [OR (95% CI): 1.29 (0.98 to 1.70), *p*-value =0.07]. In the current study, we also found that different definitions of prediabetes were associated with more than 40% risk of hypertension only among individuals aged <60 years.

Concerning CKD, a systematic review and meta-analyses, including nine cohort studies, found that excess risk associated with IFG (as FPG 110–125 mg/dl) was about 10% greater compared to those with normoglycemia, and the effect modification of age was not reported (12). In the present data set, we found that IFG status was associated with about 40 % increased risk of CKD among the younger population only in the age and sex-adjusted analysis no such associations were found among elder ones. Similarly, Vieira et al. (17), in a post hoc analysis of SPRINT trial among 9361 participants (aged ≥50 years) without diabetes, found that IFG at baseline was not associated with worsening of kidney function or albuminuria; moreover, no subsequent effect modification according to age was observed. Suzuki et al. (54), among 1 849 074 participants, showed that prediabetes (as defined by HbA1c levels of 5.7–6.4%) increased the risk of proteinuria among both participants aged < 50 and those 50 years or older.

As reported by ADA, Metformin intervention is as effective as lifestyle changes, especially among the younger population aged 25–44 years (44, 45). In the review article by Herman. W (55), the cost-effectiveness of the diabetes prevention program was evaluated. It was shown that lifestyle modification was cost-saving in individuals <45 years but cost-effective in all ages. Metformin therapy was cost-effective in participants aged < 65 years; this reduction was largely associated with its reduced effectiveness in older participants (55). Therefore, besides considering several risk factors such as BMI, or age, the risk to benefit of individualized interventions should also be considered by policy health makers. Following the current study, we observed that prediabetes was associated with a higher risk of hypertension, CVD, non-CV, and total mortality for individuals aged <65 years; regarding T2DM, associations were observed for all age groups. According to the World Health Organization STEPwise approach surveillance (WHO STEPS) surveys in Iran (2016), the prevalence of prediabetes (only using FPG criteria) was 35% in individuals aged ≥65 years (data not shown). With respect to 2016 Iranian census, of a total of 4,871,518 Iranian aged ≥65 years, 1,705,031 adults were potentially at higher risk of prediabetes, however, this population might not be at higher risk of for unhealthy outcomes, excluding T2DM, hence active surveillance of Iranian elder population with prediabetes might not justify.

### Strengths and limitations

The strengths of our analyses include the use of a large and well-designed prospective cohort study in the Middle East and North Africa. Moreover, we used accurate information on FPG, 2 h-PCG, and measured rather than self-reported confounders. However, our study has several limitations. First, HbA1c was not measured in the TLGS, which could have helped categorize prediabetes more accurately. Secondly, because of the limited number of outcomes, we did not consider different definitions of prediabetes. Thirdly, death cause-specific analysis was not possible due to the small number of events across glycemic categories for each age group. Fourthly, we did not check whether the risk for clinical outcomes is due to mild elevation in blood glucose levels or solely future development of diabetes. Finally, this study was done in the metropolitan of Tehran; therefore, it might not be generalizable to the rural zone.

## Conclusion

The high prevalence of prediabetes particularly among the elderly population, limited resources, and the observed significant age differences in the impact of prediabetes states on different clinical outcomes among the Tehranian population calls for multicomponent intervention strategies by policy health makers, including lifestyle and possible pharmacological therapy, with the priority for the young Iranian population. Meanwhile, additional work is needed to show whether the risk for clinical outcomes among the younger population is reduced by reversion to normoglycemia.

## Data availability statement

The raw data supporting the conclusions of this article will be made available by the authors, without undue reservation.

## Ethics statement

The studies involving human participants were reviewed and approved by the Institutional Review Board (IRB) of the Research Institute for Endocrine Sciences (RIES), Shahid Beheshti University of Medical Sciences. The patients/participants provided their written informed consent to participate in this study.

## Author contributions

SA and FH conceived and planned the study. SA and DK conducted the analyses. SA, SM, and FH developed the first draft of the manuscript and critically revised the manuscript. All authors contributed to the article and approved the submitted version.

## Conflict of interest

The authors declare that the research was conducted in the absence of any commercial or financial relationships that could be construed as a potential conflict of interest.

## Publisher's note

All claims expressed in this article are solely those of the authors and do not necessarily represent those of their affiliated organizations, or those of the publisher, the editors and the reviewers. Any product that may be evaluated in this article, or claim that may be made by its manufacturer, is not guaranteed or endorsed by the publisher.

## Abbreviations

MI: Myocardial infarction
FPG: Fasting plasma glucose
2 h-PCG: 2-h post-challenge plasma glucose
CVD: Cardiovascular disease
CKD: Chronic kidney disease
TLGS: Tehran Lipid and Glucose Study
NCDs: Non-communicable diseases
T2DM: Type 2 diabetes mellitus
FH-DM: Family history of diabetes
Cr: Creatinine
TC: Total cholesterol
eGFR: Estimated glomerular filtration rate
BMI: Body mass index
IFG: Impaired fasting glucose
IGT: Impaired glucose tolerance
SBP: Systolic blood pressure
DBP: Diastolic blood pressure
CHD: Coronary heart disease
HR: Hazard ratio
CI: Confidence interval
NFG: Normal fasting glucose
NGT: Normal glucose tolerance.
