# Supplementary material for: Differences in the impact of impaired glucose status on clinical outcomes in younger and older adults: Over a decade of follow-up in the Tehran lipid and glucose study
Source: Front Cardiovasc Med. 2022 Oct 31;9:1018403. doi: 10.3389/fcvm.2022.1018403 (PMC9662168; doi:10.3389/fcvm.2022.1018403)
Supplement: Supplementary file 1 [file Data_Sheet_1.docx]

| **Table S1 : Adjusted hazard ratios (95 % CI) for incident HTN considering ACC/AHA definition by age groups: Tehran Lipid and glucose study** | | | | | | | | |
| --- | --- | --- | --- | --- | --- | --- | --- | --- |
|  | **Age <60 years** | | |  | **Age ≥60 years** | | |  |
|  | **E/N** | **Model 1** | **Model 2** |  | **E/N** | **Model 1** | **Model 2** | **Interaction** |
|  |  | **HR (95 % CI)** | **HR (95 % CI)** |  |  | **HR (95 % CI)** | **HR (95 % CI)** | **p-value*** |
| **Incident HTN** | | | | | | | |  |
| FPG <100 mg/dl | 1118/3656 | Reference | Reference |  | 145/429 | Reference | Reference | 0.73 |
| FPG (100-125 mg/dl) | 29/100 | 0.99(0.81-1.17) | 0.97(0.81-1.17) |  | 9/22 | 1.05(0.72-1.54) | 1.05(0.71-1.53) |  |
| 2h-PCG <140 mg/dl | 1002/3337 | Reference | Reference |  | 120/357 | Reference | Reference | 0.32 |
| 2h-PCG (140-199 mg/dl) | 145/419 | **1.22(1.02-1.47)** | **1.23(1.02-1.48)** |  | 34/94 | 0.99(0.66-1.47) | 0.99(0.66-1.47) |  |
| FPG <100 mg/dl or 2h-PCG <140 mg/dl | 1090/3601 | Reference | Reference |  | 136/406 | Reference | Reference | 0.61 |
| FPG (100-125 mg/dl) and 2h-PCG (140-199 mg/dl) | 57/155 | 1.29(0.98-1.70) | 1.29(0.97-1.70) |  | 18/45 | 1.10(0.67-1.83) | 1.11(0.67-1.83) |  |
| FPG: fasting plasma glucose; 2h-PCG: 2-hour post-challenge plasma glucose; HR: hazard ratio; T2DM: type 2 diabetes mellitus; HTN: hypertension; CKD: chronic kidney disease.  Model 1: adjusted for age, and gender; model 2: adjusted for age, gender, body mass index, hypercholesterolemia, current smoking, eGFR, prevalent CVD  * In multivariable model | | | | | | | | |

| **Table S2: Adjusted hazard ratios (95 % CI) for incident CVD and CV mortality by age groups for population with insulin information: Tehran Lipid and glucose study** | | | | |
| --- | --- | --- | --- | --- |
|  |  | **Age <60 years** |  | **Age ≥60 years** |
|  | **E/N** | **HR (95 % CI)** | **E/N** | **HR (95 % CI)** |
| **Incident 1st CVD (n=2,996)** | | | |  |
| FPG <100 mg/dl | 125/2246 | Reference | 71/307 | Reference |
| FPG (100-125 mg/dl) | 31/351 | 1.33(0.88-2.00) | 30/92 | 1.23(0.78-1.94) |
| 2h-PCG <140 mg/dl | 137/2341 | Reference | 80/326 | Reference |
| 2h-PCG (140-199 mg/dl) | 19/256 | 1.08(0.64-1.80) | 21/73 | 1.01(0.60-1.70) |
| FPG <100 mg/dl or 2h-PCG <140 mg/dl | 146/2497 | Reference | 90/365 | Reference |
| FPG (100-125 mg/dl) and 2h-PCG (140-199 mg/dl) | 10/100 | 1.67(0.87-3.20) | 11/34 | 1.00(0.48-2.07) |
| **CV mortality (n=3,129)** | | | |  |
| FPG <100 mg/dl | 8/2300 | Reference | 8/353 | Reference |
| FPG (100-125 mg/dl) | 2/367 | 1.20(0.25-5.73) | 5/109 | 2.42(0.75-7.76) |
| 2h-PCG <140 mg/dl | 9/2393 | Reference | 11/371 | Reference |
| 2h-PCG (140-199 mg/dl) | 1/274 | 1.00(0.12-8.00) | 2/91 | 0.91(0.19-4.24) |
| FPG <100 mg/dl or 2h-PCG <140 mg/dl | 9/2558 | Reference | 13/418 | Reference |
| FPG (100-125 mg/dl) and 2h-PCG (140-199 mg/dl) | 1/109 | 2.38(0.29-19.41) | 0/44 | - |
